# 

**Published:** 1891-04

**Authors:** 


### CONTENTS:

Article I.—The Odontological Society of Great Britain, - 529

" II.—The Obtunding of Sensitive Dentine. By George F. Eames, M. D., D. D. S. - - 545

" III.—The Treatment of Dead Teeth. By E. Balding, 551

" IV.—Dental Medicaments with Special Reference to Antiseptics.—By J. W. Jungman, D. D. S., - 562

" V.—The Dental Pulp. By W. Xavier Sudduth, A. M., M. D., D. D. S., - - - 567

### EDITORIAL.

Missouri State Dental Association, - - - 570

Baltimore College of Dental Surgery, - - - - 572

University of Maryland, Dental Department, - - 573

Atkinson Resolutions.—St. Louis Dental Society, - - 575

The Florida State Dental Association, - - - 575

Minnesota State Dental Association, - - - - 576

The Physiological Action of Sulphaldehyde, - - 576

